# Travel pattern-based bus trip origin-destination estimation using smart card data

**DOI:** 10.1371/journal.pone.0270346

**Published:** 2022-06-24

**Authors:** Inmook Lee, Shin-Hyung Cho, Kyoungtae Kim, Seung-Young Kho, Dong-Kyu Kim

**Affiliations:** 1 Innovative Transportation and Logistics Research Center, Korea Railroad Research Institute, Uiwang-si, Gyeonggi-do, Republic of Korea; 2 Department of Civil and Environmental Engineering, Seoul National University, Seoul, Republic of Korea; 3 Department of Transportation Engineering, University of Seoul, Seoul, Republic of Korea; 4 Institute of Construction and Environmental Engineering, Seoul National University, Seoul, Republic of Korea; Instituto de Fisica Interdisciplinar y Sistemas Complejos, SPAIN

## Abstract

Smart card data are widely used in generating the origin and destination (O–D) matrix for public transit, which contains important information for transportation planning and operation. However, the generation of the O–D matrix is limited by the smart card data information that includes the boarding (origin) information without the alighting (destination) information. To solve this problem, trip chain methods have been proposed, thereby greatly contributing in estimating the destination using the smart card data. Nevertheless, unlinked trips, that is, trips with unknown destinations, are a persisting issue. The purpose of this study is to develop a method for estimating the destination of unlinked trips, in which trip chain methods cannot be applied, using temporal travel patterns and historical boarding records of the passengers based on long-term smart card data. The passengers were clustered by k-means clustering, and the time-of-day travel patterns were estimated for each cluster using a Gaussian mixture model. The travel patterns were formulated to estimate the destination of the passengers from the smart card data. The proposed method was verified using the 2018 smart card data collected in Sejong City, South Korea. The existing trip chain method matched the destinations of 60.0% of the total trips, whereas the proposed method improved the matching to 74.9% by additionally matching the destinations of 37.2% of the unlinked trips.

## Introduction

Smart card data contain the usage and operation records of public transportation, and are comprehensive and widely used alternatives to the sampling data from public transportation surveys. Several studies have been conducted on the analysis of public transportation status using smart card data. Moreover, further studies, such as destination estimation using data mining processes, have been actively proposed. Previous studies have noted the advantages of smart card data for public transportation, as follows:

- Records on the use of public transit can be continuously collected [1].
- Compared with public transportation surveys, it can be used to analyze the behavior of passengers on a larger scale over a longer period [2].
- Because accurate time stamps and geotags are stored in the transactions (trip records), accurate information can be obtained temporally and spatially [3].

Despite these advantages, smart card data have several limitations in providing public transit information. In particular, as smart card data are mainly related to public transportation fares charged to the users and their distribution among the operators, they include basic boarding records but lack information, such as destination (alighting stop) and trip purpose. Thus, the data cannot be properly applied to public transportation planning. Even in Korea, where smart cards are commonly used, there is insufficient information on the alighting point of the passengers, except in areas that use fare plans, such as Seoul, where it is necessary to acquire the alighting stop information.

The destination information in smart card data is essential for estimating the origin and destination (O–D) matrix. Transportation planners can analyze the public transportation status through various methods using the O–D matrix, such as checking the distribution of the demand for public transit, analyzing the congestion in vehicles, and calculating the distance traveled. In addition, an O–D matrix is important for public transit operations and planning as it can be used for line design or operation planning optimization. Several studies have been conducted to estimate the passenger destination to generate an O–D matrix using smart card data.

**Fig 1:**
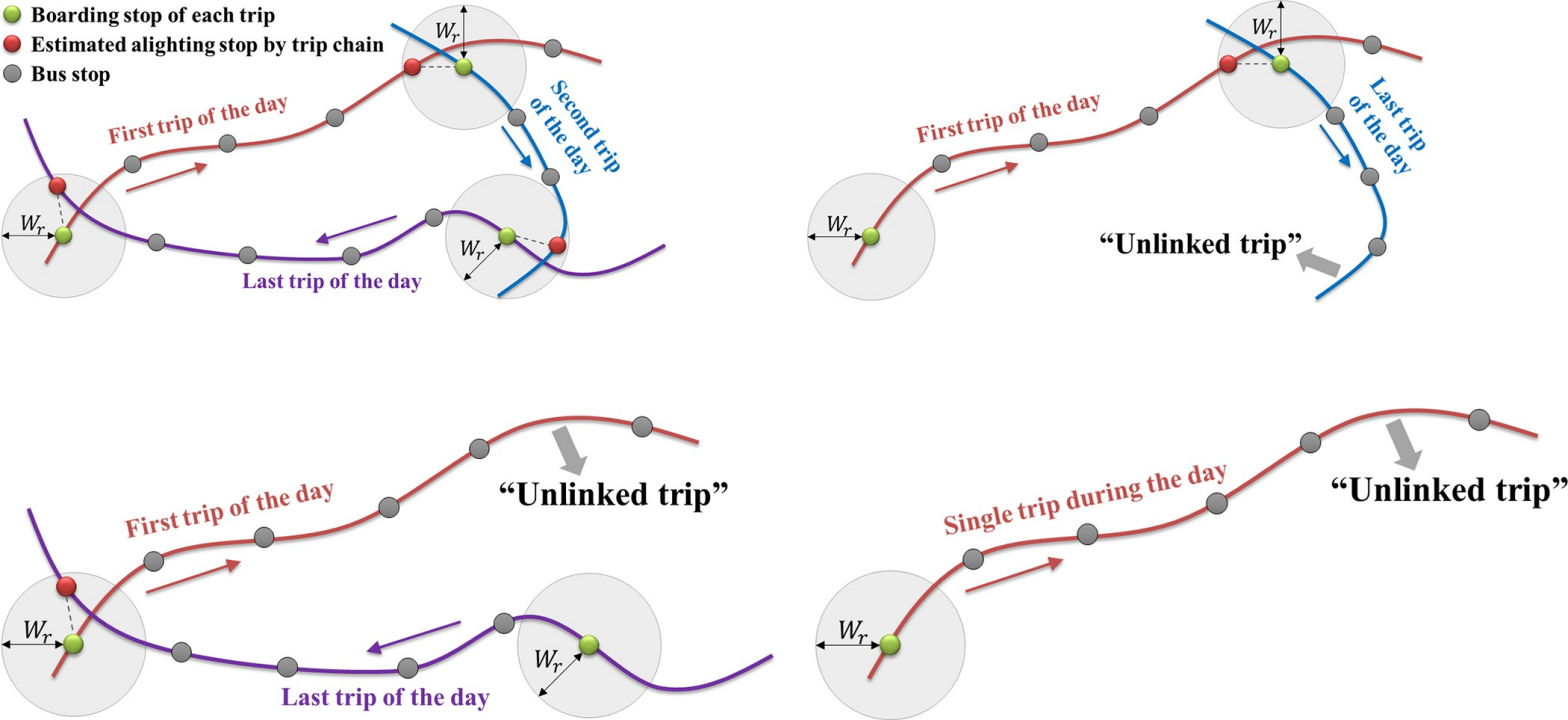
Trip chain method and cases of unlinked trips.

The trip chain method proposed by Barry et al. [4] is the most widely used method for estimating the destination of passengers using smart card data. The trip chain method estimates a destination using a relatively simple method but it cannot be applied to the estimation of unlinked trips, which are not considered as a part of a trip chain during the day. Fig 1 shows the concept of the trip chain method and the cases of unlinked trips. The alighting stop of a trip is estimated by linking one of the stops constituting the trip to the boarding stop of the next trip (or the first trip of the following day) and considering the allowable walking distance *W*_*r*_ (Fig 1A). However, if there are no links, such as the last trip in Fig 1B or first trip in Fig 1C, the alighting stops cannot be estimated. In addition, in the case of a single trip, as shown in Fig 1D, the alighting stop cannot be estimated.

This study developed a method for estimating the destination of unlinked trips (as shown in Fig 1B–1D) by incorporating the temporal travel patterns of public transit use obtained by longitudinally utilizing several days of smart card data. We clustered the passengers based on their historical boarding records and applied a mixture model to each cluster to generate travel patterns to estimate the destinations. Unlike the current trip chain methods that use the next and first trips of the day (or the following day) as the references for the estimation [4, 5], the proposed method used historical travel records collected during several days as the references to estimate the destinations of unlinked trips.

This paper is organized as follows: the subsequent section presents a review of related studies on destination estimation and travel pattern analysis using smart card data. Next, the methodologies for generating the travel patterns and estimating the destinations are described. Subsequently, the verification of the developed model using the smart card data of Sejong City in Korea is presented. The improvement in the destination estimation of unlinked trips, which is a problem with the existing trip chain method, is evaluated. Finally, the conclusions and suggestions for future work are presented.

## Literature review

O–D estimation is an important process for estimating the trip generation of each O–D pair in transportation planning and operation. Several estimation methods have been developed for O–D matrix generation. The trip chain method has been applied for the O–D estimation process of each passenger using smart card data [4–6]. However, the estimation of unlinked trips is a challenge when generating a full O–D matrix. This section focuses on previous studies related to trip destination estimation and passenger clustering using smart card data.

### O–D estimation

The trip chain method, which is a representative method for estimating the trip destinations using smart card data, was first proposed by Barry et al. [4]. This method arranges the trips of each passenger with respect to the boarding time. If there is a stop within a certain distance from the next boarding stop among the stops constituting the current boarding route, the stop is estimated as a destination. Two assumptions were made to estimate the destination after the construction of the trip chain: 1) the boarding location of most passengers coincides with the alighting location of the previous trip, and 2) the destination of the last trip of the day is the first boarding location of the daily trip for most of the passengers [4]. The concept of maximum walking distance has been introduced to the trip chain method [5, 7–9]. The maximum walking distance refers to the threshold of the distance between the alighting location of the current trip and boarding location of the next trip; distances of 400 m to 2 km have been suggested [5, 8–10]. Consequently, a method for chaining trips using the transfer time has been proposed. Consecutive boarding trips of 30–90 min were linked to a trip chain [11–13].

In addition, trips on other days were referred to sample cases, wherein it was impossible to construct the trip chains within one day [5]. The trip chain method cannot be applied to a single trip because of the lack of linkage to other trips. Thus, the generalized time was introduced to improve the accuracy of the estimation and to estimate a stop, which considered the destination, based on the minimum generalized travel cost (sum of the in-vehicle travel time and transfer walking time) among the potential alighting stops [6]. The in-vehicle time was calculated based on the bus schedule at the boarding stop to improve the accuracy of the generalized time [14]. The changes in the estimation accuracy according to the change in the maximum walking distance and transfer time (threshold of the transfer decision) were presented [15]. The estimation accuracy of the trip chain method was measured using the smart card data, including the alighting information, wherein the optimal and most accurate maximum walking distance was found to be 500 m [16]. In addition to the trip chain method, some studies have applied learning-based methods for destination estimation. The temporal and spatial alighting probability of each stop was calculated using historical travel records to estimate the destination of unlinked trips [17]. A deep learning model was developed to estimate the destinations using smart card and land use data [18]. In addition, a deep learning approach was applied to generate personalized travel patterns and predict successive points of interest [19].

There are two types of conventional trip chain methods: quantitative methods, which increase the matching percentage by adding rules (or assumptions) for the chain linking, and qualitative methods, which improve the accuracy of the estimation. Recent studies have estimated the O–D matrix using new methods, such as applying a probability model to the trip chain method or completely reorganizing the methodology with a deep learning model. This paper proposes a method for estimating the destination of unlinked trips using smart card data, which increases the matching percentage of the trips using historical trip records from a quantitative point of view and improves the estimation accuracy using the temporal travel characteristics of the passengers from a qualitative point of view.

### Passenger clustering

Recently, several studies have used smart card data to understand the travel patterns and behaviors of passengers [20–24]. This study also analyzes the travel pattern of each transit passenger to estimate their trip destination. As it is difficult to practically plan the public transportation operations according to the travel characteristics of each passenger, it is reasonable to group the passengers with similar travel characteristics and use them for planning and operation. The existing studies that analyzed the travel characteristics by clustering the temporal travel characteristics of the passengers are reviewed below.

In previous studies, partitioning methods represented by k-means are mainly used to cluster passengers using smart card data [13, 20, 25]. Some studies have applied hierarchical clustering methods [26], whereas others have applied the k-means method in combination with other clustering methods. After using k-means, the travel pattern was analyzed by applying the hierarchical ascendant classification [25]. Alternatively, a complex method of analyzing the travel regularity with the k-means and a clustering method based on DBSCAN for recognizing patterns were applied [13].

Several studies have approached clustering using model-based methods. Model-based clustering is employed to recover the original model from a dataset [27], which is suitable for modeling and analyzing data characteristics, such as travel patterns. Mixture models are mainly used as a model-based clustering method, where passengers with similar temporal travel patterns are clustered by a unigram mixture model or Gaussian mixture model (GMM) [3, 28–30]. Furthermore, a model combining k-means and GMM was applied to simultaneously cluster passengers and trips within each cluster [3, 29, 30]. This study applies clustering methods based on the temporal travel characteristics necessary for estimating the destination of the passengers and classifies the travel types using smart card data.

## Methodology

Under the premise that adequate historical travel records (boarding location and time) are available, the following were assumed for estimating the destinations of unlinked trips. First, it was assumed that a passenger’s destination is near a stop where he/she has frequently boarded. Second, it was assumed that the destination is dependent on the departure (boarding) time. Based on these two assumptions in this study, the destination of a passenger was defined as any one stop (or nearby) that is related to the boarding time of the trip among the stops from where the passenger has boarded with high frequency in the past. For example, for a passenger with a general commuting pattern, if a trip that occurred in the morning is regarded as a work-bound trip, there is a high probability that the destination is near the location where the passenger has boarded frequently in the evening, that is, it is inferred as the location of the office. Likewise, if a trip that occurred in the evening is regarded as a home-bound trip, it is considered that the destination is likely near the location where the passenger boarded frequently in the morning, that is, it is inferred as the location of the home. This study extended the time range for searching the reference trips compared to conventional trip chain methods, in which the reference trip is determined in the order of the next trip, first trip of the day, and first trip of the following day for estimating the destination.

To estimate the destination of a trip based on these assumptions, it is necessary to first analyze the characteristics of a passenger’s boarding time, which are useful for estimating the destination. In this paper, “trip pattern” is defined as “the probability of trip generation at the boarding time of the day,” and density function (distribution) of the probability of trip generation. The method of generating the trip patterns of the passengers consists of two steps: generating the travel profiles and extracting the travel patterns.

### Travel profile generation

Because public transit is utilized only a limited number of times during a day, we attempted to analyze the travel characteristics by accumulating the usage records for several days longitudinally. It is necessary to set an appropriate time interval to count the trip frequency because the boarding time is recorded in seconds. That is, the smaller the time interval is, the lower the probability of counting the trips for each time bin is. In contrast, as the size of the time interval increases, the probability of aggregation increases but the expression of the travel characteristics may be oversimplified. El Mahrsi et al. [3] measured the distribution of the variability of the number of trips observed in stations for time bins ranging from 1 min to 12 h. They observed that the variability decreases as the size of the time bin is increased. Meanwhile the mobility patterns become more predictable and apparent, and the variability decreases significantly when increasing the size of the time bin from 1 min to 1 h. Thereafter, the decrease becomes less pronounced. Consequently, they considered 1-h time bins based on their apparentness and relevance of the mobility patterns [3], which was also considered in our study. We generated personal travel profiles by counting the trip records within the analysis period at time intervals of an hour for each passenger. Considering the operating hours of public transportation, the time range was set from 5 am to 11 pm. Accordingly, the travel profile of each passenger was expressed as a vector (***c***_***i***_) composed of 19 components, as shown in Eq (1). As the number of trips varies for each passenger, the travel profile was generalized by modifying it in the form of the ratio of the number of trips within the time bin to the total number of trips.

$$c_{i}=(c_{i}^{1},c_{i}^{2},c_{i}^{b},\cdots ,c_{i}^{B})$$

$$p_{i}^{b}=\frac{c_{i}^{b}}{\sum_{b=1}^{B}c_{i}^{b}}$$

$$u_{i}=(p_{i}^{1},p_{i}^{2},p_{i}^{b},\cdots ,p_{i}^{B}),$$ (1)

where ***c***_***i***_ is the travel profile of passenger *i*, $c_{i}^{b}$ is the total number (count) of boarding trips in time bin *b* of passenger *i* during the analysis period, *B* is the number of time bins (19 components), *u*_*i*_ is the travel profile of passenger *i*, and $p_{i}^{b}$ is the probability of the trip occurrence in time bin *b* for the total number of trips of passenger *i*.

**Fig 2:**
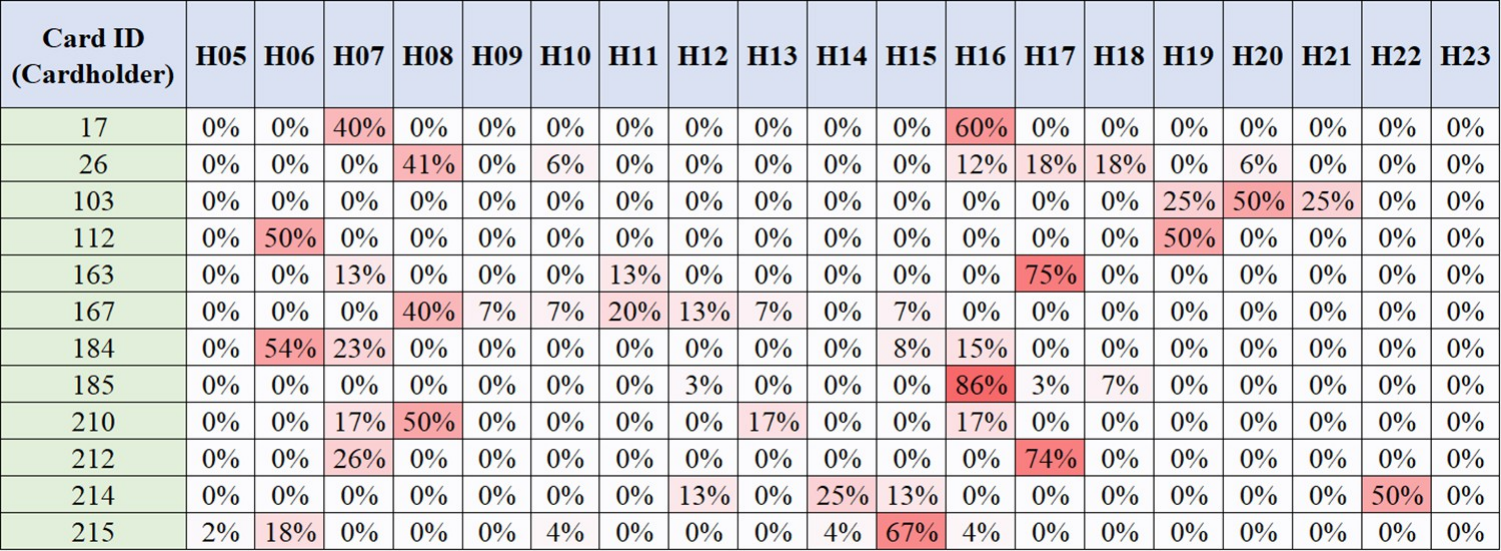
Travel profile (sample). Note: The columns represent the time bins; for example, H08 is the ratio of the number of trips per hour from 8:00 a.m. to 9:00 a.m. to the total number of trips of a passenger. The rows contain the data for each passenger, and the number of rows in the travel profile table matches the number of passengers included in the dataset.

Fig 2 illustrates the travel profiles of each passenger. When generating a travel profile, it is necessary to identify the transfer trips. To prevent duplicating the number of trips, only the representative trip (first trips) among the single-purpose trips linked by the transfers should be used to generate the travel profile. For example, a passenger departs at 8:10 a.m., makes the first transfer at 8:30 a.m., and makes the second transfer at 8:50 a.m. In this example, if the transfer trips are not identified, the number of trips between 8:00 a.m. and 9:00 a.m. is counted as three. By identifying the transfer trip, the number of trips can be corrected to one trip. Thus, we identified the transfer trips based on temporal and spatial assumptions. If the difference between the boarding time of the previous trip and current trip is within the allowable time (*t*^*F*^), and the distance between the alighting point of the previous trip and boarding point of the current trip is within the allowable walking distance (*W*_*r*_), the current trip is determined to be a transfer trip. In this study, we assumed the thresholds for the transfer trips considering the size and structure of the city, that is, *t*^*F*^ was set to 1 h, and *W*_*r*_ of 500 m was applied, as proposed by Kim and Lee [16]. However, the allowable walking distance cannot be applied to the identification of the transfer trip because the smart card data do not have information on the alighting point. As an alternative, if there is a stop whose distance from the boarding point of the current trip is within the allowable walking distance among all the stops on the previous route, the current trip is considered to meet the spatial transfer criterion. The formula is as follows:

$${\mathrm{Assume}\;“T}_{i,j+1}{”\;\mathrm{as}\;a\;\mathrm{transfer}\;\mathrm{trip}\;\mathrm{of}\;“T}_{i,j}”$$

$$\mathrm{when}\;(x_{i,j+1}-x_{i,j})\leq t^{F}\;\mathrm{and}\;\{z|d^{E}(z,O_{i,j+1})\leq W_{r}\}\neq \emptyset$$

$$T_{i,j}=\{O_{i,j},\;x_{i,j},\;R_{i,j}\},$$ (2)

where *T*_*i*,*j*_ is the passenger *i*’s *j*-th trip; *O*_*i*,*j*_ is the boarding point of *T*_*i*,*j*_; *x*_*i*,*j*_ is the boarding time of *T*_*i*,*j*_; *R*_*i*,*j*_ is the route of *T*_*i*,*j*_; *z* is a potential alighting point of *T*_*i*,*j*_ (*z* is a stop along the route of *T*_*i*,*j*_ and occurs after *O*_*i*,*j*_); and *d*^*E*^(*a*, *b*) is the Euclidean distance between locations a and b. In this study, the Euclidean distance was applied to the calculation of the walking distances because of the limited pedestrian network data. However, the use of the available pedestrian network data allows a more precise calculation of the transfer distance.

Based on the viewpoint that a travel pattern is generated by the repetition of trips, it is necessary to set a minimum condition for the number of days the public transportation is utilized during the analysis period. We generated the travel profiles for those who used public transportation for more than four days, which was approximately 10% of the analysis period of 38 days. Therefore, passengers who used public transportation for less than four days during the analysis period were excluded from the travel profile and pattern generation owing to insufficient data for extracting their travel patterns. As counting the trips based on the time bins may cause a boundary problem, it is necessary to use the data from several days to mitigate this issue.

### Travel pattern extraction

**Fig 3:**
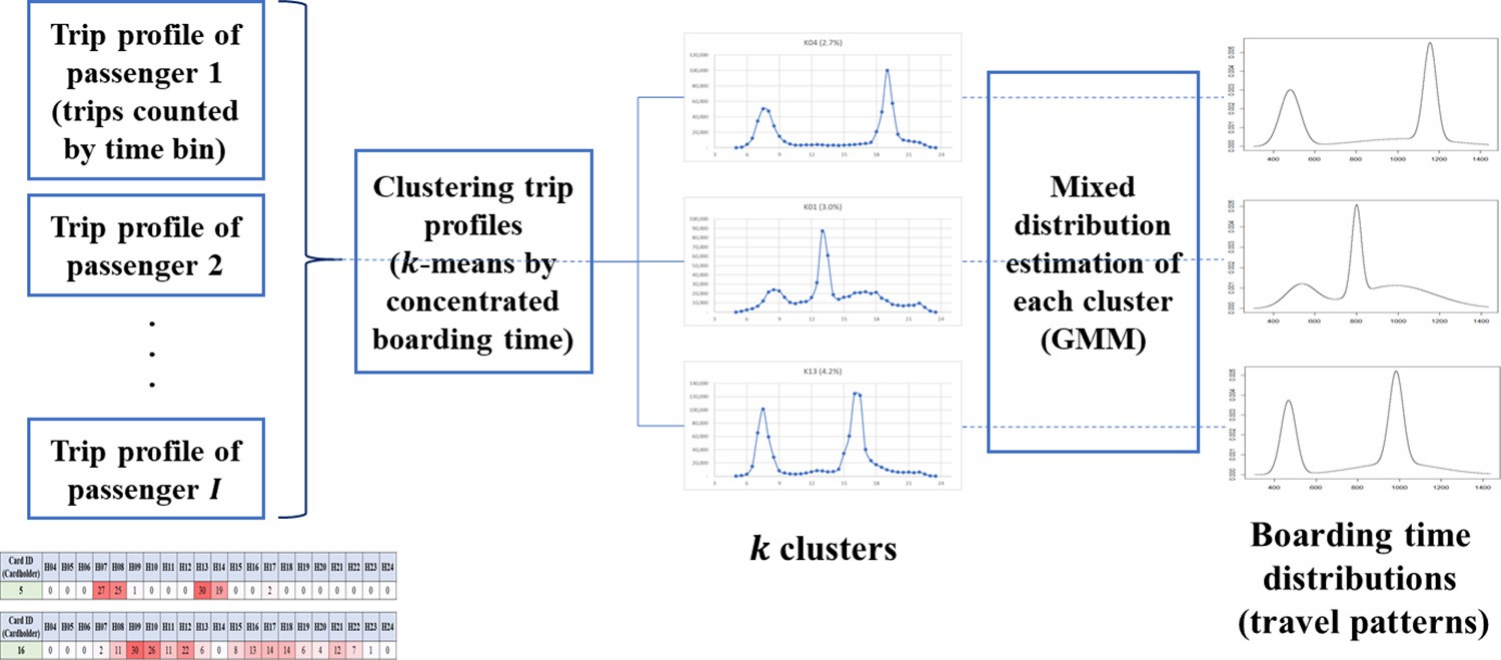
Concept of travel pattern extraction. Note: Travel patterns are extracted by clustering each passenger’s trip profile and estimating mixed distributions (GMM) of the clustered profiles.

As the next step in generating the travel profile, the generated individual travel profiles were clustered into *K* clusters according to the similarity in the travel patterns. Briand et al. [30] clustered the travel profiles using GMM. We modified the model to perform behavior analysis of public transit usage. Briand et al. [30] extracted the travel patterns, that is, the density functions of the boarding time, using a two-step generative model. First, the travel profiles were clustered into *K* clusters, and then the distribution of each cluster was estimated using a GMM. The estimated distribution of each cluster is a polynomial distribution in the form of a mixture of *H* Gaussian distributions. Similarly, this study employed two steps: the clustering of the profiles and GMM estimation. However, the method of applying the solution of the algorithm was different. Briand et al. [30] fixed both the number of clusters and number of Gaussians before applying the solution; thus, all clusters had the same number of Gaussians. As the number of Gaussians, which is the distribution of the concentrated boarding time, is also a characteristic of each cluster, we modified the method so that the number of Gaussians was different for each cluster. To apply this concept, we first clustered the travel profiles. Subsequently, the GMM was estimated for each cluster. Fig 3 shows the process of generating the travel profiles and patterns through clustering and GMM estimation. The first step (clustering) was used to generate macro-travel patterns using the aggregated data by time bins, whereas the second step (GMM estimation) was applied to estimate the boarding time distributions of each cluster using the original time data.

This study applied the partitioned clustering method by considering the data characteristics of the travel profile set, which contains large and high-dimensional variable data. As a similarity measurement method, the Euclidean distance, which is the most commonly used approach for clustering time series data [27], was applied. k-means, k-median, and k-medoid meet these clustering algorithm and similarity measurement conditions. As the computation time is proportional to the square of the number of samples, k-median and k-medoid are not suitable for application to large-scale data; therefore, k-means was chosen as the clustering method.

Clustering by k-means is suitable for large-scale and high-dimensional data. In this study, the number of clusters was determined using the elbow method, which is a heuristic method. Considering the homogeneity of the travel profile within the cluster, the total sum of squared errors (SSE) for each cluster according to the increase in the number of clusters was measured, and the point (elbow) at which the change in this value slowed down was chosen as the number of clusters. Because the clustering object comprised the travel profiles of the passengers, the cluster to which each passenger belonged was determined to be the output of the clustering. Therefore, the cluster numbers *k*_*i*_, *k*_*i*_∈{1, 2, ⋯, *K*}, were added to each passenger and each trip as follows:

$$T_{i,j}=\{O_{i,j},\;x_{i,j},\;R_{i,j},\;k_{i}\}.$$ (3)

The GMM can be expressed as follows:

$$Z_{i,j}|k_{i}=1∼Μ(1,\tau_{k,h}),$$

$$x_{i,j}|Z_{i,j}k_{i}=1∼N(\mu_{k,h},\sigma_{k,h}).$$ (4)

Eq (3) suggests that trip *j* of passenger *i* belonging to cluster *k*_*i*_ follows a polynomial distribution consisting of a mixture of *h*, *h*∈{1,⋯,*H*_*k*_} distributions. *Z*_*i*,*j*_ is a latent variable, and the parameter *τ*_*k*,*h*_ is the proportion of each distribution. Eq (4) indicates that the boarding time *x*_*i*,*j*_ of trip *j* of passenger *i* follows one of the *H*_*k*_ Gaussian distributions constituting cluster *k*_*i*_ to which passenger *i* belongs. The mean and standard deviation of each Gaussian distribution are *μ*_*k*,*h*_ and *σ*_*k*,*h*_, respectively. The parameters were estimated using the expectation maximization algorithm, which is a traditional solution of the mixture model. The Gaussian mixture distributions were estimated for each cluster, and the Gaussian distribution number *h* to which the trip belonged was added to each trip.


$$T_{i,j}=\{O_{i,j},\;x_{i,j},\;R_{i,j},\;k_{i},\;h_{i,j}\}$$ (5)

**Fig 4:**
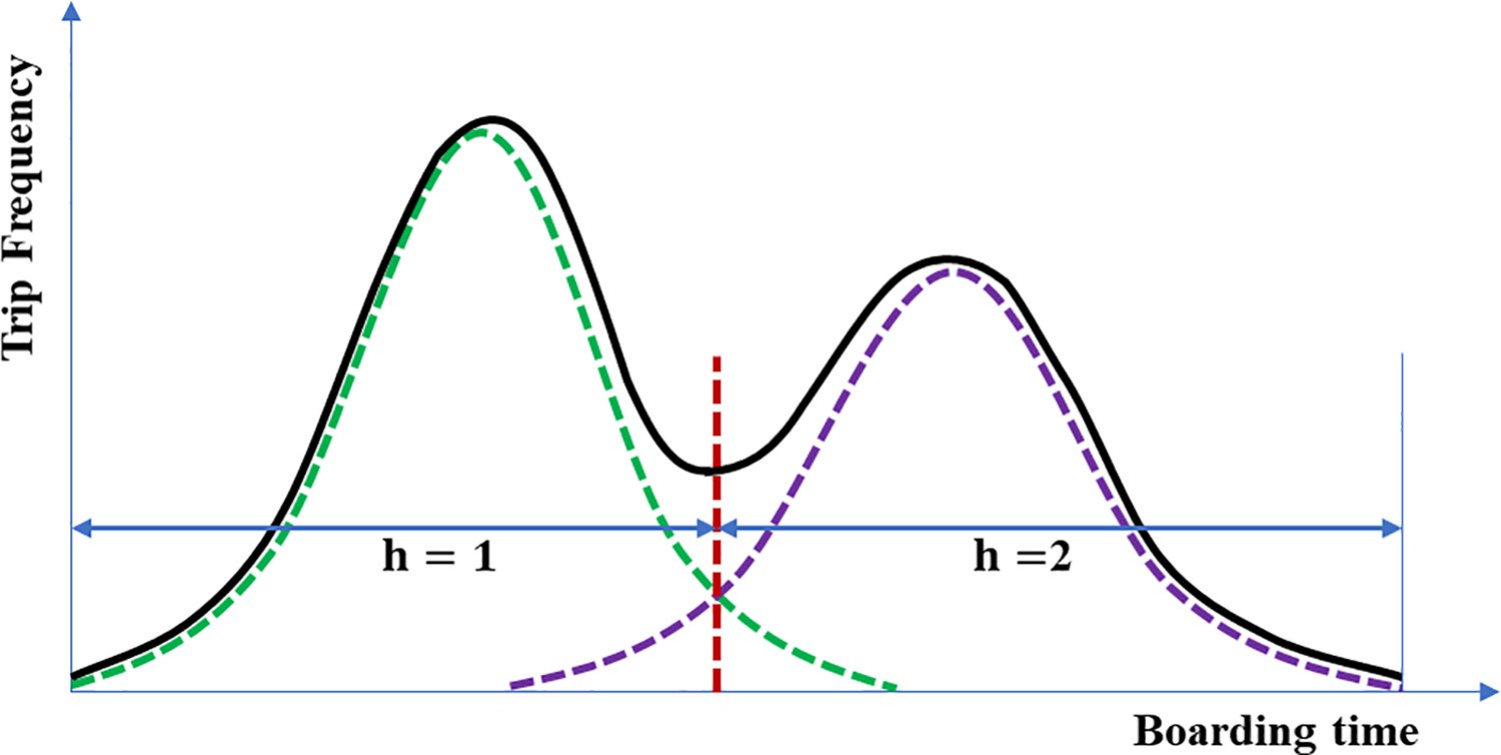
Example of a trip pattern consisting of a mixture of two Gaussians.

**Fig 5:**
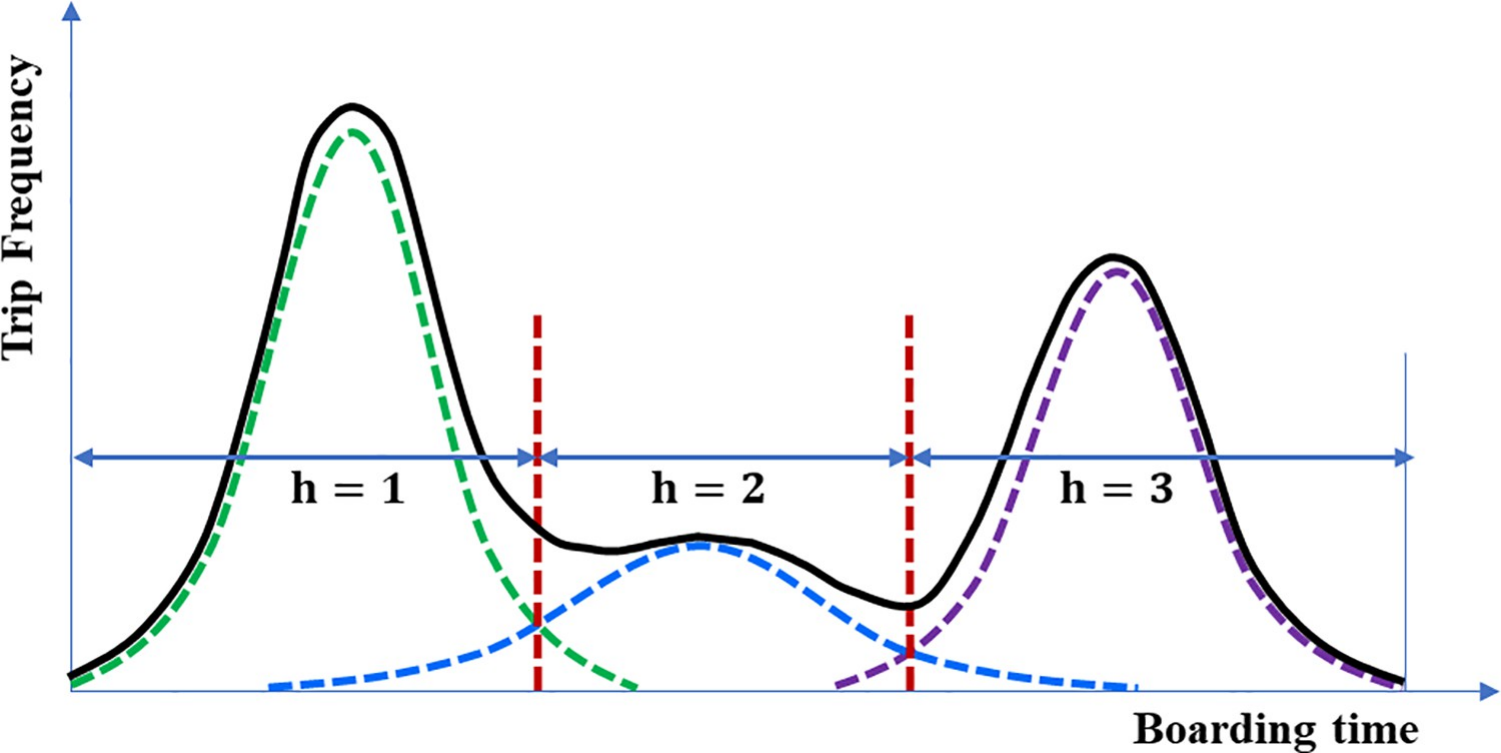
Example of a trip pattern consisting of a mixture of three Gaussians.

*H*_*k*_, which is the number of Gaussians for each cluster, is also a parameter that must be set like the number of clusters *K*. We estimated the Gaussian mixture distribution of the boarding times of the trips belonging to the cluster by changing *H*_*k*_ in the range of 1 to *N* for each cluster. The Gaussian number *H*_*k*_ was chosen according to the integrated completed likelihood criterion. Through the GMM estimation, the distribution of the boarding times of the passengers belonging to each cluster was estimated, as shown in the examples in Figs 4 and 5.

As a result, the travel pattern can be represented by *H*_*k*_ sets of Gaussian parameters (*τ*_*k*,*h*_, *μ*_*k*,*h*_, *σ*_*k*,*h*_). Given the boarding time *x*_*i*,*j*_ of a certain trip, the probability of that trip for each Gaussian distribution can be calculated using the Gaussian parameters and probability equation of the Gaussian distribution. The most probable distribution is assigned to *h*_*i*,*j*_, which is the Gaussian distribution to which the trip belongs.


$$h_{i,j}=\underset{h\in \{1,\cdots ,H_{k}\}}{\arg\;max}\frac{1}{\sigma_{k,h}\sqrt{2\pi }}exp\;(-{\left(x_{i,j}-\mu_{k,h}\right)}^{2}/2\sigma_{k,h}^{2})$$ (6)

### Destination estimation

The trip chain method estimates the destination of a trip by referring to other trips of that passenger on the same day or on nearby dates. Unlike the traditional trip chain method, our proposed destination estimation method estimates the destination of a trip by referring to trip records and travel patterns of the past days, months, or years. This section describes the process of referring to historical trip records for the destination estimation.

As seen in Figs 4 and 5, time sections can be created from the GMM estimation according to the temporal characteristics of the trips (two time sections in Fig 4 and three time sections in Fig 5). After generating the travel patterns, the time section to which all the trips of the passengers belong was assigned to the passengers who satisfy the condition for extracting the travel patterns. In this study, the condition was that the passenger must have used public transit for more than a total of four days. As mentioned at the beginning of this section, it was assumed that the destination of a trip depends on the departure (boarding) time. By applying this assumption, we assumed that the destination of a trip depends on the time section of the boarding time to which the trip belonged. In addition, according to the basic assumption of this study, the locations from where a passenger frequently boards can be potential destinations for that passenger. However, if the historical boarding records in the same time section are referred to as the destinations for trips boarded in a time section, a problem may arise where the origin and destination are the same location. Therefore, the destination of a trip in a time section (*h*) was estimated by referring to the historical boarding records in other time sections (h*, h*≠*h*), i.e., excluding the analyzed time section.

**Fig 6:**
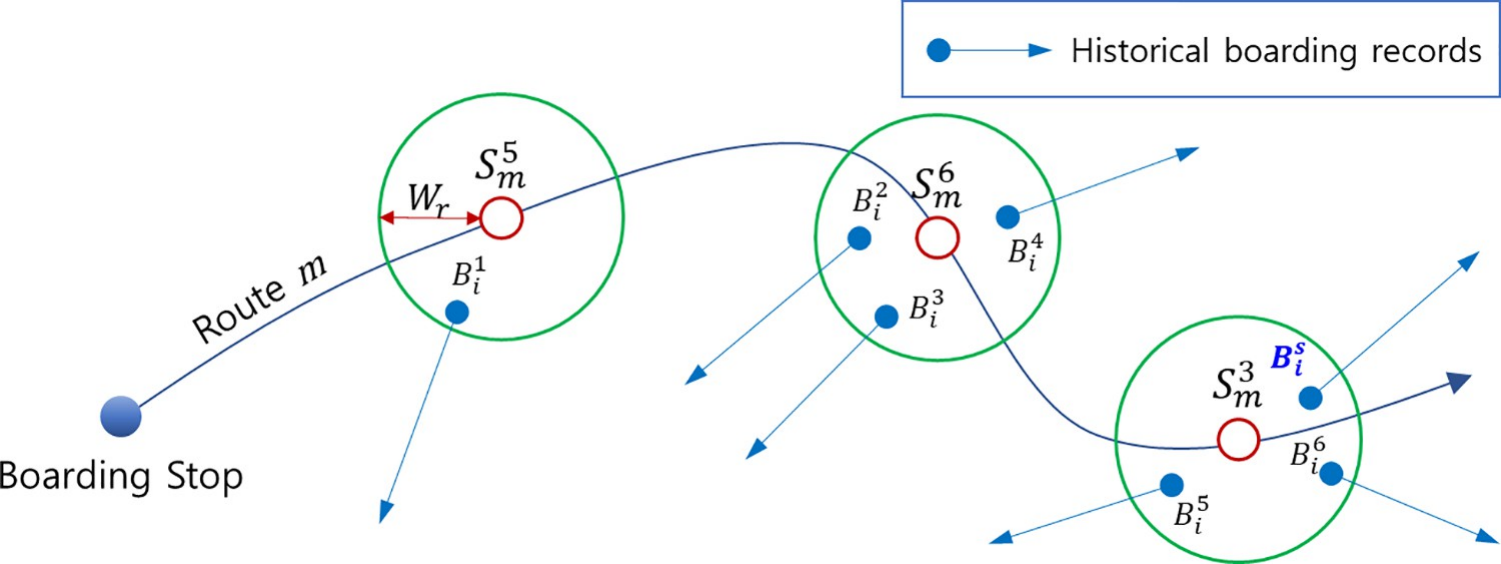
Concept of generating the historical trip (boarding) records. Note: $S_{m}^{q}$ is the ***q***-th stop along route ***m***, $B_{i}^{s}$ is the **s**-th historical boarding stop of passenger ***i***, and ***W***_***r***_ is the nearby radius (500 m in this study).

He and Trépanier [17] stored the historical records of the passengers’ alighting points on a trip route as potential destinations. They developed the density functions of the alighting probability according to the alighting frequency and time concentration for each potential destination, and estimated the stop with the highest probability as the destination. We examined the use of historical records of potential destinations by He and Trépanier [17], and employed this concept to estimate the destination based on previously described travel patterns. However, this paper proposes a method using the historical boarding records without the historical alighting records. Using the route information of the trip to be estimated, the nearby stops and boarding frequency at each stop were stored for each of the stops along route *m*. We applied *W*_*r*_ of 500 m, as suggested by Kim and Lee [16], as the nearby radius. Fig 6 shows the concept of generating the travel records for each passenger.

**Fig 7:**
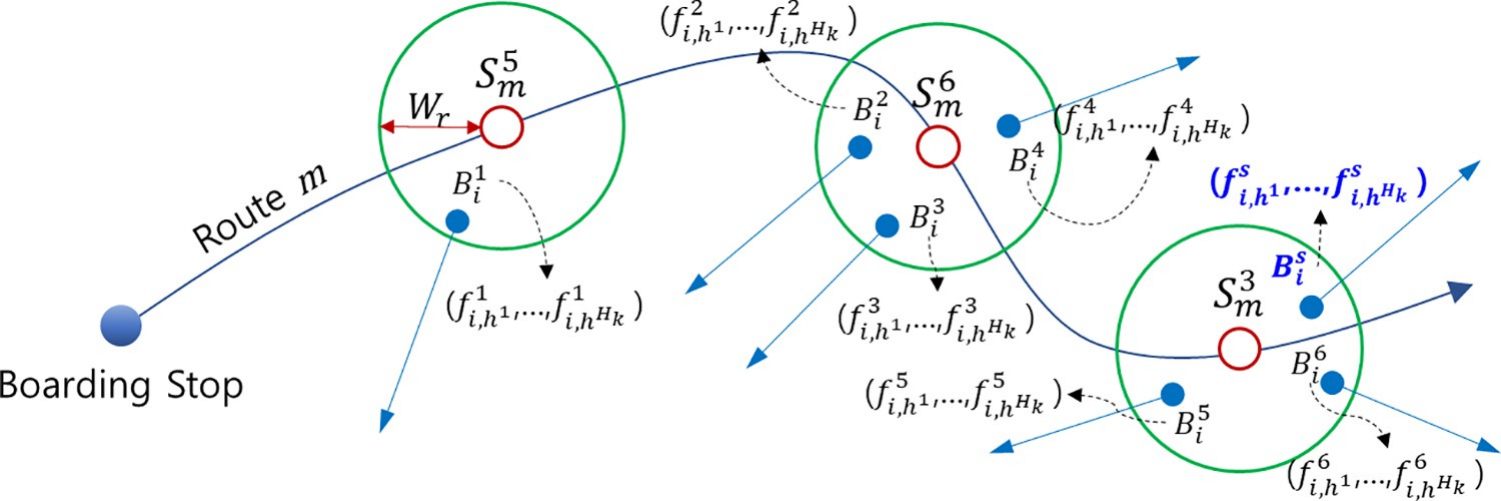
Concept of generating the historical trip records using the travel pattern as references. Note: $f_{i,h}^{s}$ denotes the historical boarding frequency at stop $B_{i}^{s}$ in the time section ***h***, ***h***∈(**1**,⋯,***H***_***k***_}.

To correlate the travel patterns with the historical boarding records, the latter were collected for each Gaussian distribution number *h* corresponding to each time section. As shown in Fig 7, if a passenger follows a traffic pattern of a mixture of two Gaussians, a boarding record set was generated for each *h* = 1 and *h* = 2.

After the set of historical boarding records for each time section was generated for each potential stop, $p_{h}(S_{m}^{q})$, which is the alighting probability in the time section *h* of the potential stop $S_{m}^{q}$, was calculated using the historical boarding frequencies of each stop, where *q*, *q*∈{1,⋯,*Q*_*m*_}, is the stop sequence number, and *s*, *s*∈{1,⋯,*S*}, is the ID number of each stop.

$$p_{i,h}(S_{m}^{q})=\frac{\sum_{s=1}^{S}D_{q}^{s}∙f_{i,h^{*}}^{s}}{\sum_{{q=q}^{b}+1}^{Q_{m}}\sum_{s=1}^{S}D_{q}^{s}∙f_{i,h^{*}}^{s}},$$ (7)

where h* is a time section other than time section *h*; *q*^*b*^ is the stop sequence of the boarding stop of trip *T*_*i*,*j*_, (*q*≥*q*^*b*^+1); $D_{q}^{s}$ is a dummy variable indicating whether $S_{m}^{q}$ and $B_{i}^{s}$ are nearby (1: $d^{E}(S_{m}^{q},B_{i}^{s})\leq W_{r}$, 0: $d^{E}(S_{m}^{q},B_{i}^{s})>W_{r}$).

The stop with the highest probability of alighting was estimated as the destination. When there were several stops with the same probability, the stop with the smallest stop sequence was identified as the destination to prevent choosing an incorrect stop. In the case of two time sections, as shown in Fig 4, the destinations were estimated by cross-referencing. Meanwhile, for three or more time sections, as shown in Fig 5, the priority of the reference should be determined. Considering that Trépanier et al. [5] estimated the destination by referring to the first trip of the day for analyzing the last trip of the day, we estimated the destinations by primarily referring to the historical boarding records in the first time section (*h* = 1) for the trips in the second or later sections (*h*≥2). As self-referencing was not possible for the trips in the first time section, the time section with the largest *τ*_*k*,*h*_ value, which is the proportion of each time section (distribution) estimated in the travel pattern generation step to the second or later time sections, was primarily referred. After the primary reference, the trips of that section were referenced in the order of the time section with the largest *τ*_*k*,*h*_ value. For example, in the case of *τ*_*k*,*h* = 1_>*τ*_*k*,*h* = 3_>*τ*_*k*,*h* = 2_ in a travel pattern that is a mixture of three distributions, the trips in *h* = 3 and *h* = 2 refer primarily to the historical boarding records in the first time section (*h* = 1). The trips in *h* = 1 refer primarily to the historical boarding records at *h* = 3, which is the section with the largest *τ*_*k*,*h*_ value. For *h* = 1, *h* = 2, and *h* = 3, for the trips whose destinations could not be estimated in the primary reference, the historical boarding records in the remaining time sections, i.e., *h* = 2, *h* = 3, and *h* = 2, respectively, were referred.

## O–D estimation and validation results

### Data description

We verified the proposed method using the smart card data from Sejong City, which is an administrative capital city in the Republic of Korea with a population of approximately 300,000 and area of 464.9 km^2^. The data were provided by the Ministry of Land, Infrastructure and Transport (the Republic of Korea) in October 2018 for transportation-related analysis and research. The card numbers were encrypted to prevent the identification of the individual travelers. Smart card data for 42 weekdays in the period April 1 and May 31, 2018 were used. During the analysis period, 128 bus routes and 1,293 bus stops were in operation in Sejong City. The data for 38 days were used to generate the travel patterns as historical boarding records, and data for four days (from May 28 to May 31) were used for validation.

The smart card data of Sejong City consist of the transaction and operation information data. The transaction data include public transport usage records, such as card number (encrypted for privacy), transportation modes, route number, boarding stop, and boarding time. The operation information data include the route information, stop information (name and coordinates), and stop configuration for each route (set and sequence number of stops constituting the route). During the analysis period, a total of 246,516 passengers used public transportation. The data consisted of 2,253,840 bus transactions. There were 1,733,625 transaction records with the destination (alighting stop) data from the smart card data, which constituted 76.9% of the total transactions. To estimate the destinations of the transaction records using the developed method, we removed the destination data from the smart card data and re-inferred the destinations based on the boarding stop data. The destination data from the original data were only used to verify the estimation accuracy.

### Travel profile and travel pattern generation results

The original data comprised 1,614,887 trip records generated by 194,730 passengers, and 1,595,470 trips remained when the transfer trips were removed. Because the travel profiles comprise data for generating travel patterns according to the repetitions of trips, the travel profiles were generated only for passengers who had used public transportation for at least four days, which was approximately 10% of the analysis period. Consequently, we generated the travel profiles using 1,331,025 (82.4% of original data) trip records generated by 63,014 (32.4% of the original data) passengers.

**Fig 8:**
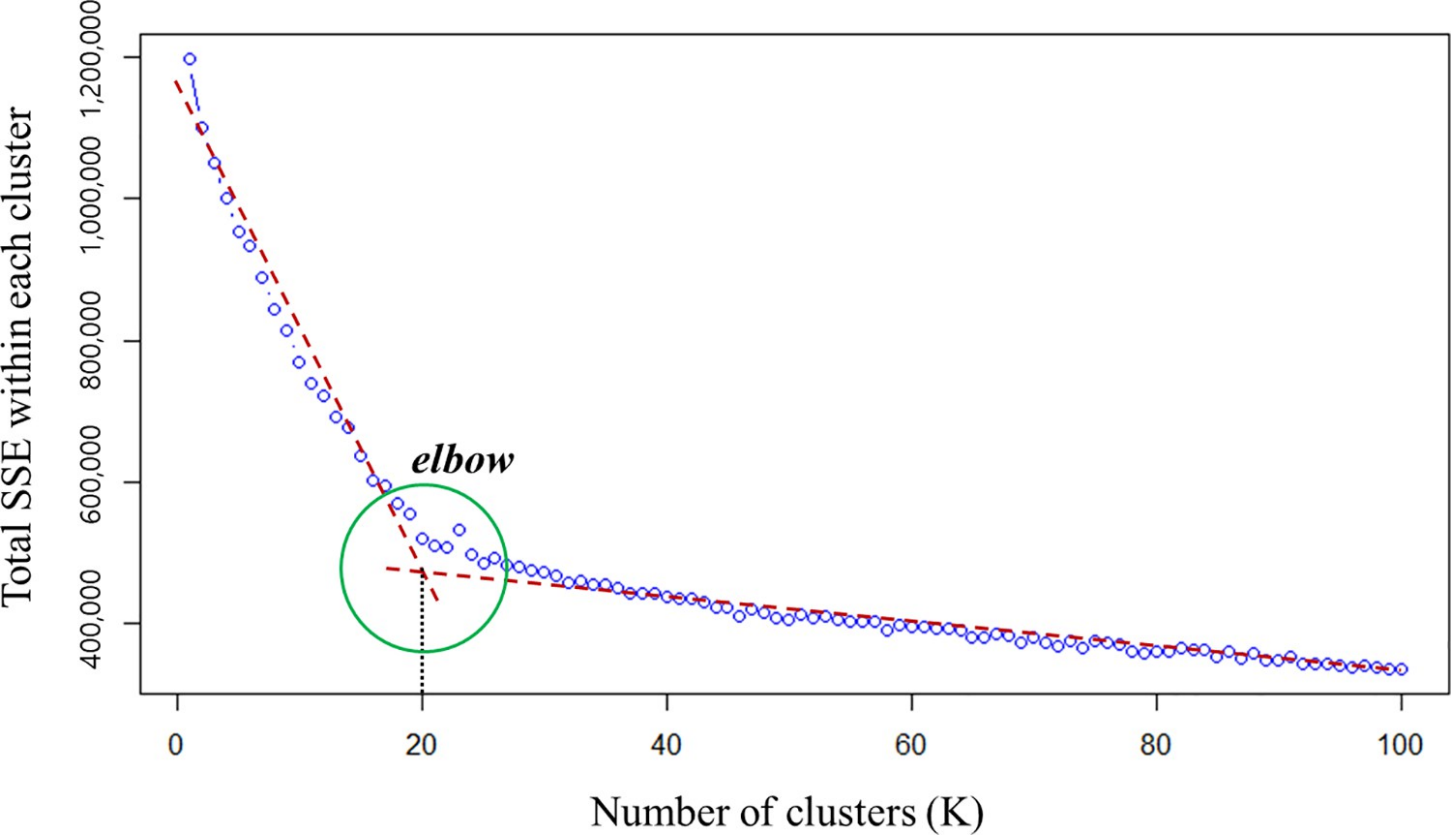
Total SSE within the cluster according to the number of clusters ***K***. Note: In this figure, an elbow is observed when the number of clusters is 20.

Before clustering the travel profiles, the number of clusters *K* and number of Gaussians *H*_*k*_ for each cluster should be determined. The number of clusters *K* was determined using the elbow method, as described above. Fig 8 shows the measurement result of the total SSE of each cluster when the number of clusters *K* was increased from 1 to 100. Clustering was considered suitable if the sum of the SSEs of each cluster decreases. When there were 20 clusters, an elbow where the error improvement efficiency clearly decreased was observed. Consequently, we clustered the travel profiles into 20 clusters; this number of clusters is only valid for the dataset in this study.

**Fig 9:**
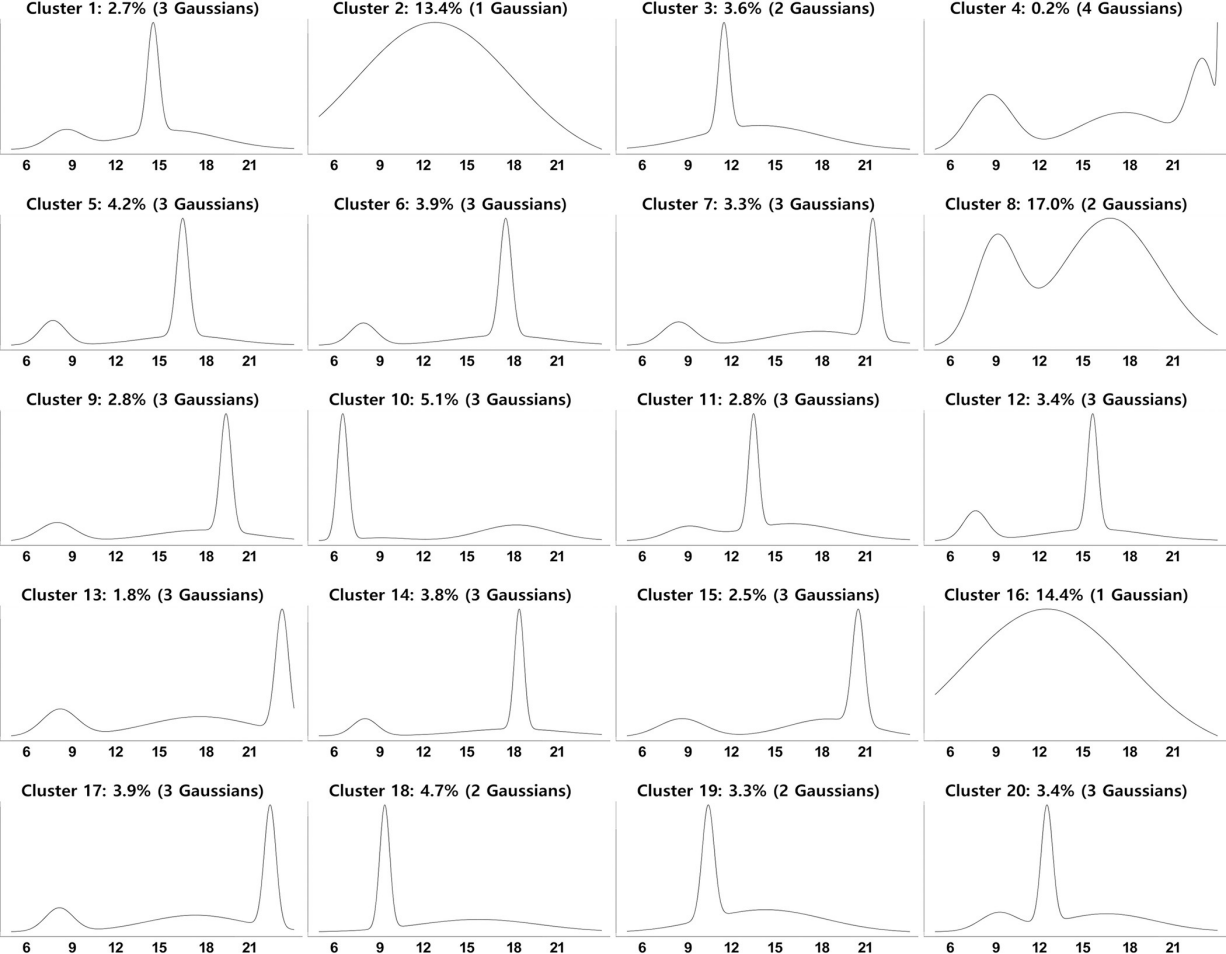
Temporal boarding time pattern of each cluster. Note: Each graph shows the boarding density over time for each of the 20 clusters.

Fig 9 shows the result of estimating the Gaussian mixture of the boarding density over time for each of the 20 clusters. Clusters 1 and 16 had one Gaussian, clusters 3, 8, 18, and 19 had two Gaussians, cluster 4 had four Gaussians, and the remaining clusters were composed of three Gaussians. The time of each Gaussian peak was *μ*_*k*,h_. The visible form of the Gaussian mixture is necessary for the qualitative analysis of the travel patterns, whereas the parameters (*τ*_*k*,h_, *μ*_*k*,h_, *σ*_*k*,h_) constituting each Gaussian are important in the quantitative analysis of the travel patterns. Hence, each travel pattern can be quantitatively expressed as a set of parameters, as listed in Table 1. Taking cluster 7 as an example, a trip belonging to cluster 7 has a 22% probability of belonging to a distribution centered at 8.46 hours (approximately 8:30), whereas the probability of belonging to a distribution centered at 21.52 hours (approximately 21:30) is 40%, and the probability of belonging to the rest of the distribution is 38%. Two peaks of the three Gaussian distributions are distinctly observed in the figure, but one of them has a relatively low and broad distribution.

**Table 1:** Gaussian mixture parameters of the travel pattern for each cluster. Note: The parameter ***τ***_***kh***_ is the proportion of each distribution, the parameter ***μ***_***kh***_ is the mean (Gaussian center) of each distribution, and the parameter ***σ***_***kh***_ is the standard deviation of each distribution.

Cluster number (*K* = 20)		*k* = 1	2	3	4	5	6	7	8	9	10	11	12	13	14	15	16	17	18	19	20
Number of Gaussians (*H*_*k*_)		3	1	2	4	3	3	3	2	3	3	3	3	3	3	3	1	3	2	2	3
*τ*_*kh*_	*h* = 1	0.17	1.00	0.28	0.25	0.22	0.20	0.22	0.26	0.22	0.51	0.16	0.23	0.20	0.19	0.23	1.00	0.18	0.45	0.33	0.18
	*h* = 2	0.34	-	0.72	0.37	0.48	0.48	0.38	0.74	0.43	0.06	0.34	0.39	0.45	0.51	0.40	-	0.43	0.55	0.67	0.36
	*h* = 3	0.49	-	-	0.21	0.30	0.32	0.40	-	0.36	0.43	0.50	0.38	0.34	0.30	0.37	-	0.39	-	-	0.46
	*h* = 4	-	-	-	0.17	-	-	-	-	-	-	-	-	-	-	-	-	-	-	-	-
*μ*_*kh*_	*h* = 1	8.64	12.79	11.51	8.70	7.79	7.98	8.46	9.09	8.07	6.60	9.04	7.71	8.26	8.10	8.69	12.51	8.22	9.42	10.45	9.26
	*h* = 2	14.53	-	13.93	17.76	16.52	17.55	17.87	16.78	19.43	9.17	13.50	15.60	17.68	18.46	18.53	-	17.37	15.73	14.30	12.52
	*h* = 3	15.76	-	-	23.02	16.72	17.39	21.52	-	17.93	18.27	16.01	15.73	23.20	18.08	20.54	-	22.40	-	-	16.54
	*h* = 4	-	-	-	24.27	-	-	-	-	-	-	-	-	-	-	-	-	-	-	-	-
*σ*_*kh*_	*h* = 1	1.16	5.25	0.36	1.51	0.87	0.88	1.07	1.43	1.20	0.36	1.35	0.81	1.18	0.83	1.56	5.60	0.94	0.34	0.40	1.23
	*h* = 2	0.38	-	4.05	3.24	0.40	0.39	3.10	3.33	0.38	1.76	0.33	0.34	3.56	0.32	2.74	-	3.27	4.03	3.97	0.38
	*h* = 3	3.26	-	-	0.90	3.00	3.22	0.37	-	3.33	2.40	3.28	3.33	0.44	3.28	0.40	-	0.40	-	-	3.26
	*h* = 4	-	-	-	0.15	-	-	-	-	-	-	-	-	-	-	-	-	-	-	-	-

### Destination estimation result

Because the destination estimation method proposed in this paper refers to the historical boarding records of time sections (Gaussians) other than the time section (Gaussian) to which the trip belongs, the destinations of the trips belonging to clusters with only one time section (*H*_*k*_ = 1) cannot be estimated. Therefore, the destination estimation method was applied to 18 clusters, except for clusters 2 and 16, which had travel patterns composed of only one time section. In addition, the proposed method cannot be applied to passengers who do not have a travel pattern owing to their low frequency of travel (defined as those who have used public transportation for less than four days in this study). The validation data comprised a total of 116,194 transactions, including the alighting information, and no logical errors in the sequence between the boarding and alighting stops were noted. These transactions can be used to verify the estimation accuracy.

The proposed destination estimation approach estimated 44.0% of the destinations. Compared with the actual alighting point, the estimation accuracy of the number of trips was 48.4%, and that of the total number of trips was 21.3% (= 44.0% × 48.4%). Because this method refers to the boarding records within the allowable walking distance (500 m) from the potential destination, the initial distance error of the method was 500 m. Therefore, one of the adjacent stops within 500 m could have been incorrectly estimated as the destination stop. Applying the “relaxed rule” to the accuracy metric, that is, the estimated alighting stop is within one stop difference (including previous and next stop) from the actual alighting stop, the accuracy of the estimated number of trips was 76.7%, and the accuracy of the total number of trips was 33.8%.

**Fig 10:**
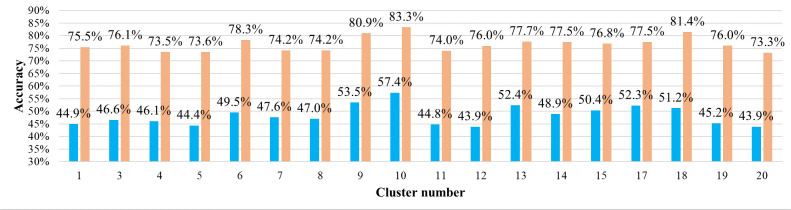
Estimation accuracies by cluster (blue: Normal rule, orange: Relaxed rule). Note: The normal rule indicates the estimated and actual alighting stops are the same, and the relaxed rule indicates the estimated alighting stop is within one stop difference (including previous and next stop) from the actual alighting stop.

**Fig 11:**
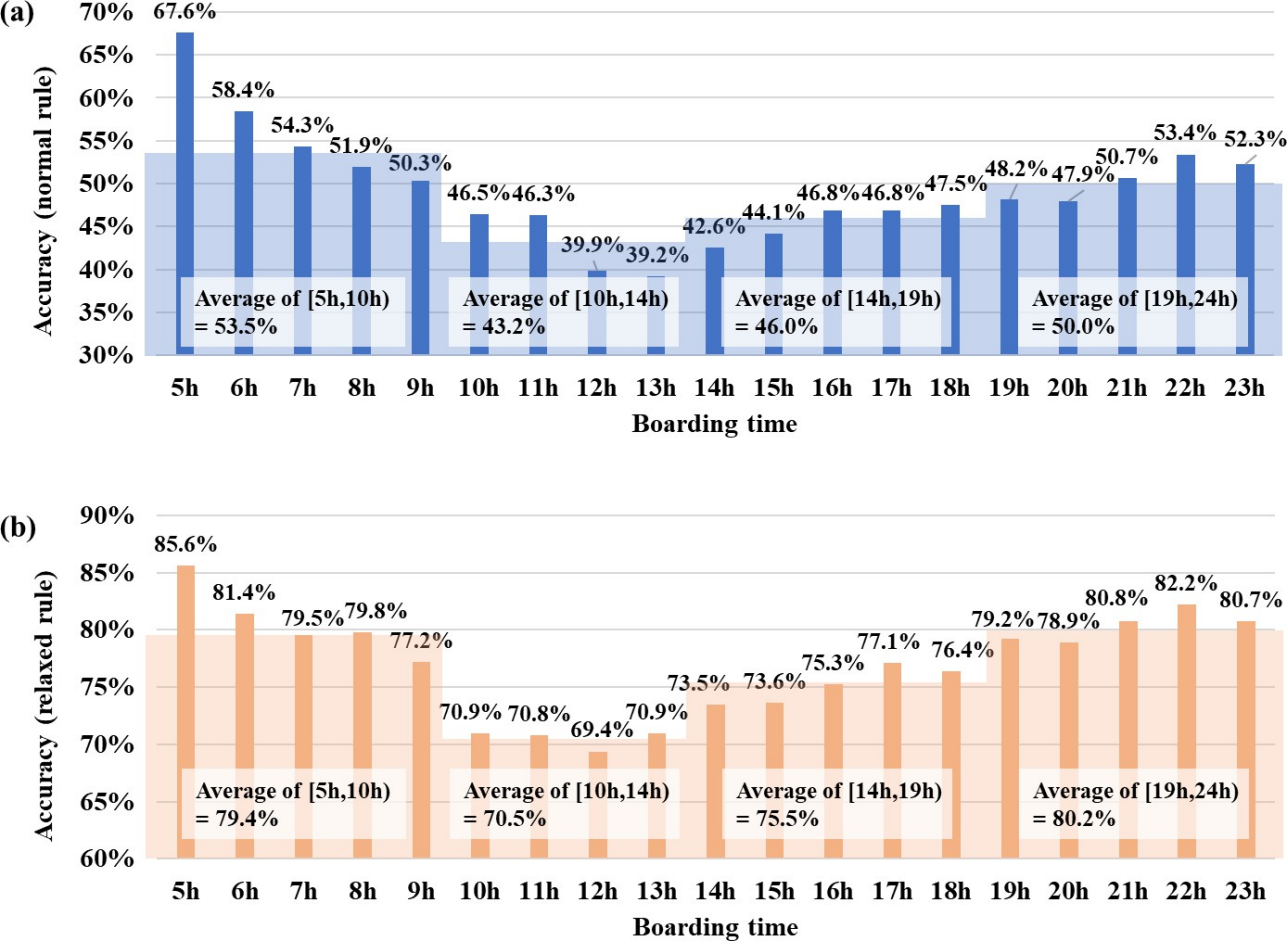
Estimation accuracies by the time range of the day (a. normal rule, b. relaxed rule).

Fig 10 shows the estimation accuracy for each cluster. The accuracy metric by the “normal rule” indicates that the estimated and actual alighting stops were the same. Table 1 summarizes the Gaussian mixture parameters for each cluster. In Table 1, *τ*_*k*,*h*_ represents the proportion of trips belonging to each time section, and *μ*_*k*,*h*_ represents the time concentration for a specific time section. The estimation accuracies of clusters 10 and 18 were relatively higher than those of the other clusters, and both clusters were characterized by high proportions of morning peak hour trips (*h* = 1), as shown in Table 1 (57.4% for cluster 10 and 51.2% for cluster 18). Therefore, our method was suitable for passengers with high concentration of trips in the morning peak hour. Fig 11 shows the estimation accuracy according to the boarding time. The boarding times of the passengers were divided into four groups according to the range of the fluctuations of the estimation accuracy. The estimation accuracies for the morning and evening trips were high, and the estimation accuracy for the daytime off-peak hours was low. This means that the morning and evening trips were more likely to follow a pattern than the daytime trips.

**Fig 12:**
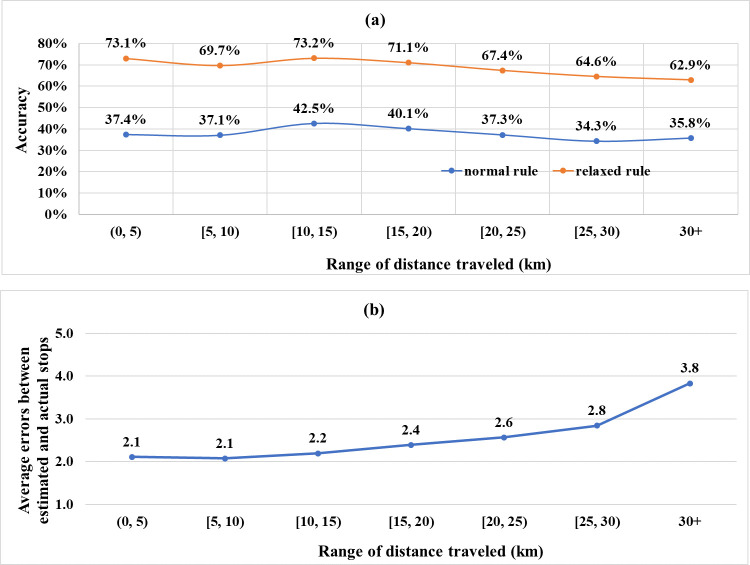
Estimation results according to the distance traveled (a. estimation accuracy, b. estimation error).

Fig 12A shows the estimation accuracy based on the distance travelled. The estimation accuracies were relatively higher for the distance of 10–15 km and remained constant up to 20 km. When the distance exceeded 20 km, the accuracies slightly decreased. Fig 12B presents the estimation errors according to the distance travelled. The numbers represent the averages of the sequential differences between the estimated and actual stops (e.g., 0 if correctly estimated). The error increased with the travel distance. Therefore, the developed method achieved a relatively good performance for trips with a travel distance of less than 20 km.

### Improvement of the current algorithm

The purpose of this study was to improve the destination estimation method of unlinked trips by referring to the historical travel patterns of the passengers. To generate a set of unlinked trips, the destination should first be estimated using the trip chain method. Trips that cannot be estimated by the chain method form a set of unlinked trips. We used the method proposed by Barry et al. [4] and applied the allowable walking distance of 500 m. Among the chosen potential destinations, the stop with the shortest generalization distance was estimated as the destination of the trip. The generalized distance [16] was calculated by adding the generalized transfer distance to the in-vehicle distance from the boarding stop to the potential stop *z* (*d*_*z*_). The generalized transfer distance was the product of the walking resistance (*f*_*w*_, 1.5 in this study), walking-to-bus speed conversion factor (*c*_*b*−*w*_, 5 in this study), and distance *d*_*z*−*post*_ from the potential stop *z* to the next boarding point.


$${Dg}_{i}=d_{z}+f_{w}c_{b-w}d_{z-post}$$ (8)

We improved the existing trip chain-based destination estimation method by applying the travel patterns to the unlinked trips, which cannot be estimated by the trip chain method. Table 2 shows the results of the destination estimation by applying the travel pattern method of this study to the set of unlinked trips generated by employing the trip chain method to the validation data (116,194 trips) in the previous section.

**Table 2:** Comparison of the estimation results by the trip chain method and improved method.

Estimation method	Trip patterns (For all trips)	Trip chain	Trip patterns (For unlinked trips)	Improved method (Trip chain + patterns)
Sample trips (validation data) (A)	116,194	116,194	46,532 (Unlinked trips)	116,194
Estimated (matched) trips (B)	51,165	69,662	17,316	86,978
Matching percentage (= B/A)	44.0%	60.0%	37.2%	74.9%
Unmatched trips (= A-B)	65,029	46,532 (Unlinked trips)	29,216	29,216
Trips with the exact match (normal rule) (C)	24,783	47,904	8,074	55,978
Accuracy for the matched trips (= C/B)	48.4%	68.8%	46.6%	64.4%
Accuracy for the total trips (= C/A)	21.3%	41.2%	17.4%	48.2%
Trips matched within one stop difference (relaxed rule) (D)	39,219	63,211	12,967	76,178
Accuracy by the relaxed rule for the matched trips (= D/B)	76.7%	90.7%	74.9%	87.6%
Accuracy by the relaxed rule for the total trips (= D/A)	33.8%	54.4%	27.9%	65.6%

When the current trip chain method was applied, the destinations of 60.0% of the trips could be estimated (matched) with the estimation accuracy of 68.8%. With respect to the total number of sample trips rather than the matched number of trips, the estimation accuracy was 41.2%. However, 46,532 trips (40.0%) were generated as unlinked trips without destination matching. By applying the travel pattern method proposed in this paper to these unlinked trips, 37.2% of the destinations could be matched, and the estimation accuracy of the matched trips was 46.6%.

When the destination was estimated by combining the trip chain method and travel pattern method, the matching percentage was improved from 60.0% to 74.9%. Although the estimation accuracy for the matched trips decreased, that for the total trips improved from 41.2% to 48.2% because the number of trips matched with high accuracy increased. As a result, accurate estimation was realized for 48.2% of the total trips and 65.6% of the trips within one stop difference. Therefore, the proposed method of estimating the destinations using the travel patterns and historical boarding records can improve the destination estimation problem of unlinked trips.

## Conclusions

In this paper, we proposed a longitudinal method that applies the temporal travel patterns and historical boarding records of public transportation passengers obtained from long-term smart card data to estimate the destinations of unlinked trips, which could not be analyzed using the trip chain method. Travel profiles were generated using the temporal frequency of the travel for each passenger. The passengers were clustered by k-means clustering. The time-of-day travel patterns were estimated for each passenger cluster using a GMM. The time sections were partitioned with the temporal characteristics of the trips, following the GMM results. Potential destinations were created to apply the temporal travel patterns to the destination estimation by aggregating historical boarding locations (stops) for each time section. Finally, a stop with high historical frequency of boarding, selected among the potential destinations in the time section to which the trip does not belong, was matched as the destination of the trip.

We applied this method to the smart card system of Sejong City, which contained alighting information that could be used for verification. As a result, 44.0% of the trips were matched. For 48.4% of the destination-matched trips, the destination was accurately estimated to the actual alighting point. Compared to existing destination estimation methods using smart card data using the trip chain method only, the proposed travel pattern method applied to the unlinked trips generated from the trip chain method increased the matching percentage by 14.9%. The number of trips with their destinations matched within the difference of one bus stop from the actual alighting stop also increased by 6.9% compared with the existing trip chain method. Therefore, the proposed method of estimating the destinations using travel patterns and historical boarding records could improve the destination estimation for unlinked trips.

However, the proposed method has some limitations. First, as the proposed method assumes a travel pattern for each passenger, the destinations of the passengers who use public transportation less frequently cannot be estimated. In particular, the destinations of the passengers who used public transportation for less than four out of 38 days, which comprised 24.6% of the total trips, could not be estimated. Second, because the destination is estimated by referring to the potential destinations belonging to the time sections other than the time section to which the trip belongs, the destinations of the trips in the clusters with a travel pattern composed of only one Gaussian cannot be estimated; the proportion of such cases was approximately 26.3%.

In the future, it will be necessary to verify whether the possibility of generating the travel patterns of the passengers can be increased by longitudinal expansion of data and to develop an estimation algorithm for trips belonging to a single Gaussian pattern. Moreover, we need to explore the developed models by considering the correlation between the spatio-temporal characteristics, including the changes in the travel patterns of the passengers over time and actual transfer distance from the pedestrian network. The improved method is expected to achieve a higher matching rate for the O–D matrix of public transportation. Although we have generated discrete travel profile data, studies that apply a continuous clustering method to continuous time data are also needed to accurately understand the travel behaviors.

## Supporting information

S1 Data (XLSX)
